# Toddler Screening for Autism Spectrum Disorder: A Meta-Analysis of Diagnostic Accuracy

**DOI:** 10.1007/s10803-018-03865-2

**Published:** 2019-01-08

**Authors:** Ana B. Sánchez-García, Purificación Galindo-Villardón, Ana B. Nieto-Librero, Helena Martín-Rodero, Diana L. Robins

**Affiliations:** 1 INICO-University of Salamanca, Pso. Canalejas, 169, 37008 Salamanca, Spain; 2 IBSAL-University of Salamanca, C/ Alfonso X El Sabio s/n, 37007 Salamanca, Spain; 3 Medical School Library, University of Salamanca, C/ Alfonso X El Sabio s/n, 37007 Salamanca, Spain; 4 AJ Drexel Autism Institute, 3020 Market Street, Suite 560, Philadelphia, PA 19104-3734 USA

**Keywords:** M-CHAT, Autism, Screening tools, Meta-analysis, Systematic review, HSROC

## Abstract

Great efforts focus on early detection of autism spectrum disorder, although some scientists and policy-makers have questioned early universal screening. The aim of this meta-analysis was to evaluate the diagnostic accuracy of the different screening tools. Several electronic databases were used to identify published studies. A Bayesian model was used to estimate the screening accuracy. The pooled sensitivity was 0.72 (95% CI 0.61–0.81), and the specificity was 0.98 (95% CI 0.97–0.99). Subgroup analyses to remove heterogeneity indicated sensitivity was 0.77 (95% CI 0.69–0.84), and specificity was 0.99 (95% CI 0.97–0.99; SD ≤ 0.01). Level 1 screening tools for ASD showed consistent statistically significant results and therefore are adequate to detect autism at 14–36 months.

**Electronic supplementary material:**

The online version of this article (10.1007/s10803-018-03865-2) contains supplementary material, which is available to authorized users.

Population level (level 1) screening for autism spectrum disorder (ASD) has been the subject of numerous papers, particularly since the American Academy of Pediatrics published a policy statement more than a decade ago (Council on Children with Disabilities 2006). The most commonly studied tool is the Modified Checklist for Autism in Toddlers (M-CHAT; Robins et al. 1999), and its revision, the M-CHAT-revised, with follow-up (M-CHAT-R/F; Robins et al. 2009). However, the variety of screening tools for prospective identification of early signs of autism has encouraged the publication of different systematic reviews (Daniels et al. 2014; McPheeters et al. 2016). See Table 1 for the tools included in the current meta-analysis, and references for more information about each tool.

**Table 1:** Details of sample characteristics and individual outcomes such as studies show

Study number	Screening test(s)	Country	*FN*^a^ strategy	*FN*	*FP*	*TP*	*TN*	*N*	Total *N*^b^	Sex		Not reported	Age (months)
										Female	Male		
1. Nygren et al. (2012)	M-CHAT	Sweden	No	NA	3	33	NA	3.985	3.999	2.087	1.912	NA	29.00
2. Nygren et al. (2012)	JOBS	Sweden	No	NA	3	37	NA	3.985	3.999	2.087	1.912	NA	29.00
3. Nygren et al. (2012)	M-CHAT + JOBS	Sweden	No	NA	5	43	NA	3.985	3.999	2.087	1.912	NA	29.00
4. Baird et al. (2000)	CHAT	UK	Yes	74	14	20	16.127	16.235	NA	NA	NA	NA	18.70
5. Wiggins et al. (2014)	M-CHAT	USA	Yes	3	17	27	3.803	3.850	3.980	NA	NA	NA	21.10
6. Wiggins et al. (2014)	PEDS+ PATH	USA	Yes	2	20	28	2.978	3.028	3.980	NA	NA	NA	21.10
7. Kamio et al. (2014)	M-CHAT_JV	Japan	Yes	22	24	20	1.661	1.727	2.141	880	847	NA	18.70
8. Stenberg et al. (2014)	M-CHAT	Norway	Yes	114	3.804	59	48.049	52.026	NA	25.429	26.597	NA	18.00
9. Chlebowski et al. (2013)	M-CHAT/Yale Screener + STAT	USA	Yes	6	79	92	18.269	18.446	18.989	9.388	9.601	NA	20.40
10. Canal-Bedia et al. (2011)	M-CHAT	Spain	Yes	0	25	6	2.024	2.055	NA	949	1.106	NA	21.40
11. Barbaro and Dissanayake (2010)	SACS	Australia	Yes	34	41	174	20.521	20.770	NA	10.177	10.593	NA	19.27
12. Inada et al. (2011)	M-CHAT (short version 9, cut-off 1)	Japan	NA	NA	NA	20	NA	1.167	1.187	571	596	NA	18.00
13. Inada et al. (2011)	M-CHAT (full version)	Japan	NA	NA	NA	20	NA	1.167	1.187	571	596	NA	18.00
14. Dereu et al. (2010)	CESDD	Belgium	Yes	13	265	28	6.502	6.808	NA	3.255	3.553	NA	16.70
15. Miller et al. (2011)	ITC + M-CHAT	USA	Yes	2	17	10	638	667	796	NA	NA	NA	NA
16. Robins et al. (2014)	M-CHAT-R/F	USA	Yes	18	116	105	15.373	15.612	16.071	7.570	7.793	249	20.95
17. Honda et al. (2005)	YACHT-18	Japan	Yes	16	NA	68	NA	35.716	NA	17.468	18.248	NA	18.00
18. Baranek (2015)	M-CHAT	USA	Yes	3	32	5	534	574	NA	300	268	6	24.73

*FN* false negative, *FP* false positive, *TP* true positive, *TN* true negative, *NA* not available from paper, *M-CHAT* modified-checklist for autism in toddlers, *JOB* joint attention-observation schedule, *CHAT* checklist for autism in toddlers, *PED* parents’ evaluation of developmental status, *M-CHAT_JV* modified-checklist for autism in Toddlers_Japanese version, *STAT* screening tool for autism in toddlers and young children, *SACS* social attention and communication study, *CESDD* checklist for early signs of developmental disorders, *M-CHAT-R*/*F* modified checklist for autism in toddlers, revised, with follow-up, *YACHT-18* young autism and other developmental disorders checkup tool

^a^*FN* strategy = methods to identify false negative screening cases, or children with ASD who were missed by the screening tool(s) of interest

^b^Total *N* with missing cases

The U.S. Preventive Services Task Force (USPSTF; Siu and Preventive Services Task Force 2016) concluded that there was insufficient evidence to provide a recommendation regarding universal toddler screening for ASD. At the same time they emphasized the potential of the M-CHAT as a universal screening tool, as evidenced by empirical results (R. Canal-Bedia, personal communication, May 9, 2016). Hence, it is necessary to perform a systematic study of the psychometric data available in different studies.

The meta-analysis is an important resource to summarize—*in quantitative terms*—the accuracy of diagnostic test, providing a higher level of evidence; for this reason, the current study conducted a meta-analysis to review empirical data from the studies and tools used since the first ASD population screening was performed in England (Baron-Cohen et al. 1996).

In this kind of study, the reference test may be imperfect because a gold standard is not available in practice. We have used the Bayesian Hierarchical Model (HSROC; Rutter and Gatsonis 2001) to carry out the meta-analysis. The model is robust in adjusting for the imperfect nature of the reference standard of autism tools, in a bivariate meta-analysis of diagnostic test sensitivity and specificity and others psychometric parameters. Another bivariate model was proposed by Reitsmaet al. (2005) in which it is assumed that the vector of (logit(sensitivity), logit(specificity)) follows a bivariate normal distribution. However, Harbord and Whiting (2009) showed that the likelihood functions of both the HSROC and bivariate models are algebraically equivalent, and yield identical pooled sensitivity and specificity. Dendukuri et al. (2012) have demonstrated the usefulness of HSROC model, when no gold standard test is available.

Therefore, in this study, we used a Bayesian meta-analysis, and the main aim was to evaluate the accuracy of the different screening tools. The second objective was to calculate the pooled psychometric properties associated with different studies to evaluate the tools effectiveness and support their recommendation internationally (R. Canal-Bedia, personal communication, May 9, 2016).

## Methods

The preferred reporting items for systematic reviews and meta-analyses (PRISMA) (Moher et al. 2009) has guided this systematic review.

### Criteria for Selection of Studies

Included papers focused on the screening and diagnosis of ASD and other developmental disorders in the general population, also known as level 1 screening. In cases where studies had duplicated data, only the most complete one was selected in order to avoid an unrealistic increase in the homogeneity between studies, and emphasis was placed on studies validating screening tools, which were often the most complete samples. Therefore, we *excluded* studies focused on tools that were not designed to screen for ASD, screening studies not applied to the general population (level 1), and all those that did not provide sufficient data to construct a 2 × 2 contingency table of screening × diagnosis (such as those without confirmatory diagnoses), or had a low quality rating in the quality assessment.

### Literature Search

A systematic literature search identified studies that reported tools and procedures used for the early detection of ASD. The articles were obtained from CINHAL, ERIC, PsycINFO, PubMed and WOS databases using several combinations of the relevant keywords and Medical Subject Heading (MeSH), which include the categories of terms suggested by Daniels et al. (2014). All articles published between January 1992 and April 2015 were considered eligible. Only articles published in the English language and reporting an age range of screening from 14 to 36 months were included. The search strategy for PubMed is described (see Appendix 1). An additional search was conducted for grey literature captured on other search engines such as Google Scholar; we also searched the reference lists of included articles and any relevant review articles identified through the search and the ‘related articles’ function in PubMed. In addition, when searching the grey literature, we took into account the reference lists of primary studies and review papers, and contacted the experts to locate significant but as yet unpublished studies.

### Assessment of Methodological Quality

Two reviewers conducted quality assessment of the included studies with the QUADAS-2 Tool (Quality Assessment of Diagnostic Accuracy Studies-2) (Whiting et al. 2004). Any discrepancies were referred to a third reviewer. QUADAS is a validated quality checklist (Deeks 2001; Whiting 2011; Whiting et al. 2006) composed of 14 items which encompass the most important sources of bias and variations observed in diagnostic accuracy studies. The studies were classified according to whether they had low or high risk for bias and their applicability was graded as low or high.

### Data Extraction

The following data items were extracted from each study using a data collection form: first author and year of publication; size and characteristics of the study population; raw cell values [true positive (*TP*), true negative (*TN*), false positive (*FP*), false negative (*FN*); and psychometric properties, specifically sensitivity (*Se*), specificity (*Sp*), positive and negative predictive values (*PPV, NPV*), positive and negative likelihood ratio values (*LR*+; *LR*−), and diagnostic odds ratio (*DOR*)]. See Appendix 2 for definitions of bio-statistical terms. Psychometric properties which were not provided in the studies were calculated based on raw cell values. Clarification was requested from the authors via e-mail when we observed discrepancies between the data reported and the data calculated. Details of the search and results are shown (see Tables 1, 2).

**Table 2:** Details of individual diagnostic outcomes such as studies show

Study	*Se*	(95% CI)	*Sp*	(95% CI)	*PPV*	(95% CI)	*NPV*	(95% CI)	*LR*+	(95% CI)	*LR*−	(95% CI)
Nygren et al. (2012)	0.767	(0.614–0.882)	NA	NA	0.917	(0.775–0.982)	NA	NA	NA	NA	NA	NA
Nygren et al. (2012)	0.860	(0.721–0.947)	NA	NA	0.925	(0.796–0.984)	NA	NA	NA	NA	NA	NA
Nygren et al. (2012)	0.956	(0.849–0.995)	NA	NA	0.896	(0.773–0.965)	NA	NA	NA	NA	NA	NA
Baird et al. (2000)	0.213	(0.130–0.300)	0.999	(0.999–1.000)	0.588	(0.420–0.750)	NA	NA	NA	NA	NA	NA
Wiggins et al. (2014)	NA	NA	NA	NA	NA	NA	NA	NA	NA	NA	NA	NA
Wiggins et al. (2014)	NA	NA	NA	NA	NA	NA	NA	NA	NA	NA	NA	NA
Kamio et al. (2014)	0.480	(0.330–0.630)	0.990	(0.980–0.990)	0.450	(0.310–0.600)	0.990	(0.980–0.990)	NA	NA	NA	NA
Stenberg et al. (2014)	0.341	(0.271–0.417)	0.927	(0.924–0.929)	0.150	(0.120–0.200)	NA	NA	4.60	NA	NA	NA
Chlebowski et al. (2013)	NA	NA	NA	NA	0.538	NA	NA	NA	NA	NA	NA	NA
Canal-Bedia et al. (2011)	1.000	NA	0.980	(0.980–0.990)	0.190	(0.050–0.330)	1.000	NA	NA	NA	NA	NA
Barbaro and Dissanayake (2010)	0.836	(0.776–0.882)	0.998	(0.998–0.999)	0.807	(0.748–0.856)	0.998	(0.998–0.999)	414.39	(303.93–564.99)	0.17	(0.12–0.22)
Inada et al. (2011)	0.650	NA	0.885	NA	0.088	NA	0.993	NA	NA	NA	NA	NA
Inada et al. (2011)	0.550	NA	0.961	NA	0.193	NA	0.992	NA	NA	NA	NA	NA
Dereu et al. (2010)	0.680	(0.540–0.830)	0.960	(0.960–0.970)	0,100	(0.060–0.130)	1.000	(0.999–1.00)	17.42	NA	0.33	NA
Miller et al. (2011)	NA	NA	NA	NA	NA	NA	0.996	NA	NA	NA	NA	NA
Robins et al. (2014)	0.854	NA	0.993	NA	0.475	NA	0.999	NA	114.05	NA	0.15	NA
Honda et al. (2005)	0.810	NA	NA	NA	NA	NA	0.999	NA	NA	NA	NA	NA
Baranek (2015)	0.625	(0.508–0.960)	0.943	NA	0.135	NA	0.994	NA	NA	NA	0.40	NA

*Se* sensitivity, *Sp* specificity, *PPV* positive predictive value, *NPV* negative predictive value, *LR*+ positive likelihood ratio, *LR*− negative likelihood ratio, *NA* not available from paper

### Data Synthesis and Statistical Analysis

We calculated the pooled *Se*, *Sp*, *LR*+, *LR*−, *PPV*, *NPV* and *DOR* for the included studies. Separate pooling of sensitivity and specificity may lead to biased results because different thresholds were used in different studies (Deeks 2001; Moses et al. 1993). Therefore, we used the Hierarchical Summary Receiver Operating Characteristic Model (HSROC) (Rutter and Gatsonis 2001) to estimate the *diagnostic accuracy* parameters and to generate a summary receiver operating characteristic curve with HSROC, [an R package available from CRAN (Schiller and Dendukuri 2015)]. The model is robust for including studies with different reference standards and potential negative correlation in paired measures (*Se*/*Sp*) across studies (Trikalinos et al. 2012). This kind of analysis models the variation in diagnostic accuracy and cut-off values, and identifies sources of heterogeneity, which is a common feature among diagnostic or screening test accuracy reviews.

The model has been called a “Hierarchical Model” owing to the fact that it takes into account statistical distributions at two levels. At the first level, within-study variability in sensitivity and specificity is examined. At the second level, between-study variability is examined (Macaskill 2004). The main goal of the model is to estimate an SROC curve across different thresholds.

The estimation from the model requires Markov Chain Monte Carlo (MCMC) simulation (Rutter and Gatsonis 2001). To carry out this Bayesian estimation we specified the prior distributions over the set of unknown parameters with a similar assumption made by Higgins et al. (2003). This process was used in order to obtain posterior predictions of the *Se* and *Sp*. According to Harbord and Whiting (2009), the true estimate of *Se* and *Sp* in each study could be found by empirical Bayes estimates, although we acknowledge that many of the included studies were limited in their ability to confirm that negative cases were in fact true negatives.

In order to establish whether there was inconsistency and heterogeneity in the meta-analysis, we summarized the test performance characteristics using a forest plot with the corresponding Higgins I^2^ index (Higgins and Thompson 2002) and assessed heterogeneity by visual inspection of the SROC plots and using Cochran’s Q test (p > 0.1) (Cochran 1954). Summary DORs were estimated by random DerSimonian–Laird effect model (DerSimonian and Laird 1986) following the recommendations of Macaskill et al. (2010) because I^2^ was greater than 50% and Q test was < 0.1. Since variability of results among different studies was confirmed, an investigation of heterogeneity was necessary and *subgroup analyses* were used. The Egger’s test (Song et al. 2002) was calculated for assessing publication bias using STATA 12.0.

Finally, we obtained a crosshair plot and ROC ellipses plot to summarize the confidence intervals of *Se* and *FP* cases in each study with the R-package (Doebler 2015) using meta-analysis of diagnostic accuracy (MADA), *LR*+, *LR*−, *PPV*, *NPV* and *DOR* were calculated using SAS for Windows, version 9.4 (Cary, NC).

## Results

### Study Selection

**Fig. 1:**
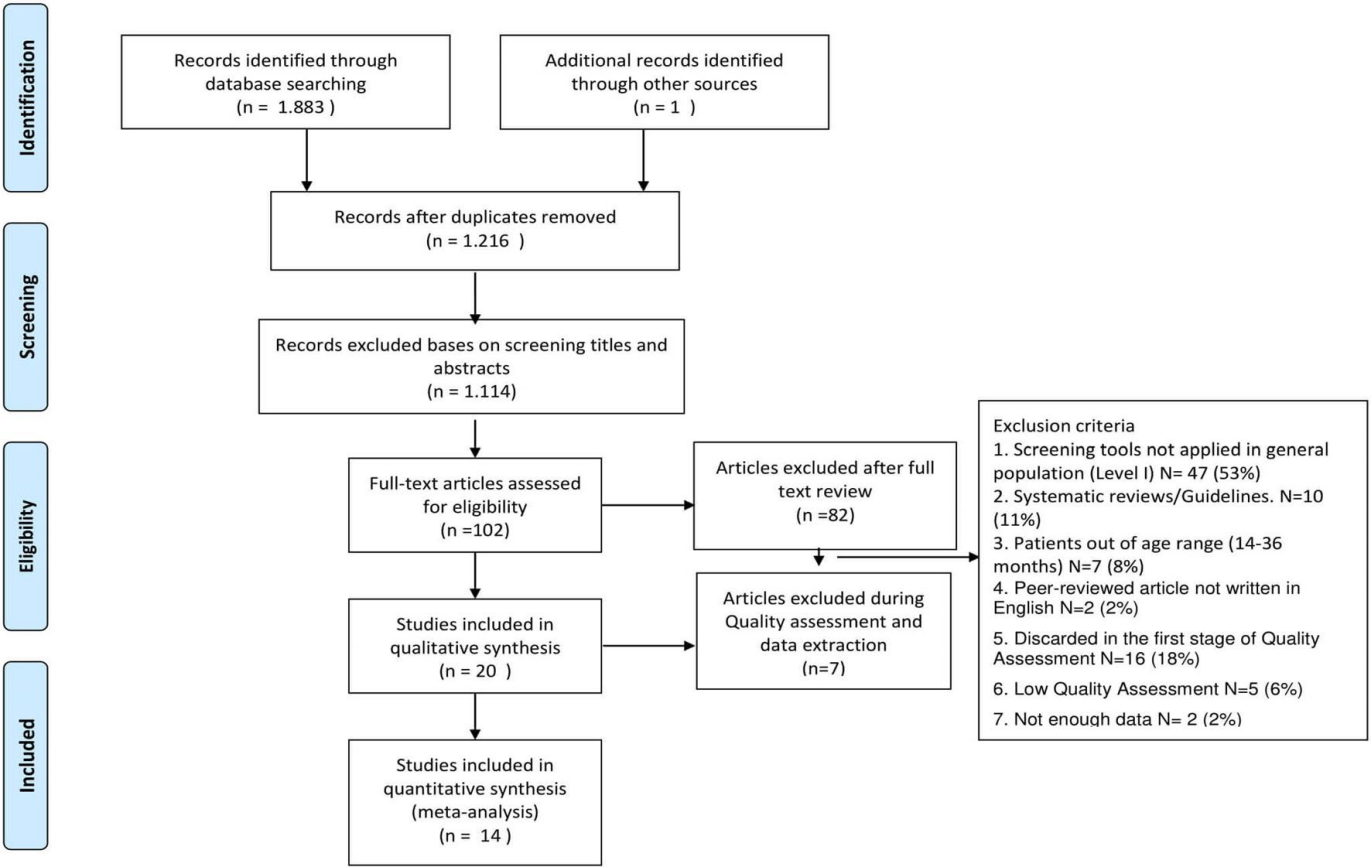
Study selection flow chart following PRISMA guidelines

The initial literature search identified 1883 studies. Six hundred and sixty-seven duplicate records were eliminated to obtain 1216 non-duplicated articles, 1114 of which were excluded after title and abstract screening through the application of inclusion/exclusion criteria, and 87 were excluded after full text screening or methodological quality assessment and data extraction (see Supplemental Table 1). One additional study that qualified for inclusion was identified from the search of grey literature. Finally, 14 studies: (Baird et al. 2000; Barbaro and Dissanayake 2010; Canal-Bedia et al. 2011; Chlebowski et al. 2013; Dereu et al. 2010; Honda et al. 2005; Inada et al. 2011; Kamio et al. 2014; Miller et al. 2011; Nygren et al. 2012; Robins et al. 2014; Stenberg et al. 2014; Wiggins et al. 2014; Baranek 2015) were eligible for inclusion in our review. We present the flow chart showing the selection process in Fig. 1.

### Methodological Quality of the Included Studies

**Fig. 2:**
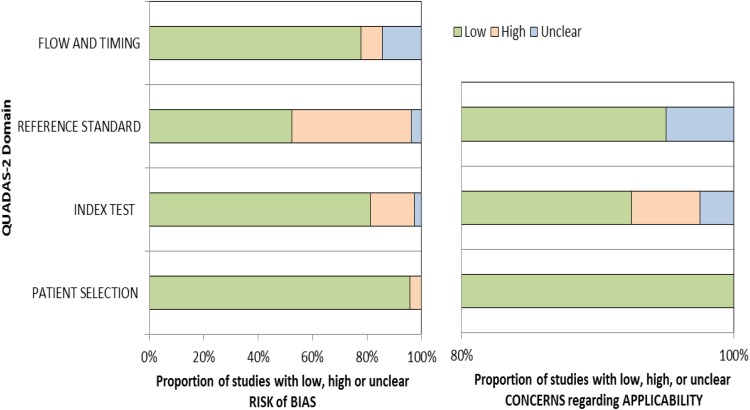
Methodological quality graph depicting the cumulative findings of the methodological quality analysis

We used the QUADAS-2 tool for study of quality assessment and K coefficient to examine inter-rater agreement for our initial overall quality score, and resolved any item discrepancies through discussion. The agreement between judges’ kappa values was 0.643 (CI 95%; p < 0.01). In Fig. 2, we summarize the results of the methodological quality for all 20 studies included in this assessment: (Baird 2000; Barbaro 2010; Canal-Bedia et al. 2011; Chlebowski 2013; Dereu 2010; Dietz 2006; Honda 2005, 2009; Inada 2011; Kamio 2014; Kleinman 2008; Miller 2011; Nygren et al. 2012; Pierce 2011; Robins 2008, 2014; Stenberg 2014; VanDenHeuvel 2007; Wetherby 2008; Wiggins et al. 2014).

As Fig. 2 shows, two bar graphs report the assessment of risk of bias and applicability. The percentage of studies rated as unclear, high, or low is observed across X-axes at intervals of 20%. The concerns regarding applicability include three domains: patient selection, index test, and reference standard. The risk of bias dimension is comprised of four domains: patient selection, index test, reference standard, and flow and timing. Across a majority of studies, concern about applicability of the reference standard was assessed as low, the index test was assessed as unclear, and patient selection was assessed as having low concerns. Regarding risk or bias, the majority of the studies demonstrated high risk of bias for flow and timing; the index test was rated as unclear risk, the reference standard was generally rated as low risk, and patient selection was rated as low risk.

During this process we excluded the following studies: Honda (2009), Pierce (2011), Robins (2008), VanDeHeuvel (2007), Wetherby (2008). In supplemental materials (see supplemental Table 1) we show the list of papers excluded during analysis of quality and data extraction processes.

### Characteristics of the Included Studies

One hundred and two full text articles were assessed for eligibility, 14 (13.72%) of which were included in the quantitative synthesis. Some articles evaluated more than one index test (Inada et al. 2011; Nygren et al. 2012; Wiggins et al. 2014) and this is why we present a meta-analysis on *18 sets* of psychometric values, 35.71% of which came from the USA, 35.71% from Europe, 21.42% from Japan and 7.14% from Australia. The sample includes 191,803 toddlers. The interval of age range is between 16.7 and 29 months. Sex data was available for 158,965 toddlers, of whom 73,431 (46.19%) were female.

The studies presented great variability in terms of the data reported. Twelve of 14 studies (66.6%) showed all the primary outcomes required to populate 2 × 2 contingency tables. Data pertaining to *Se* were presented in 77.7% of studies, *Sp* in 55.5%, *PPV* in 77.7%, *NPV* in 44.4%, and *LR*+ and *LR*− in 22.2% of studies. The main characteristics and the clinical outcomes, as shown in included studies are presented (see Tables 1, 2).

### Diagnostic Accuracy of Screening Tools

**Fig. 3:**
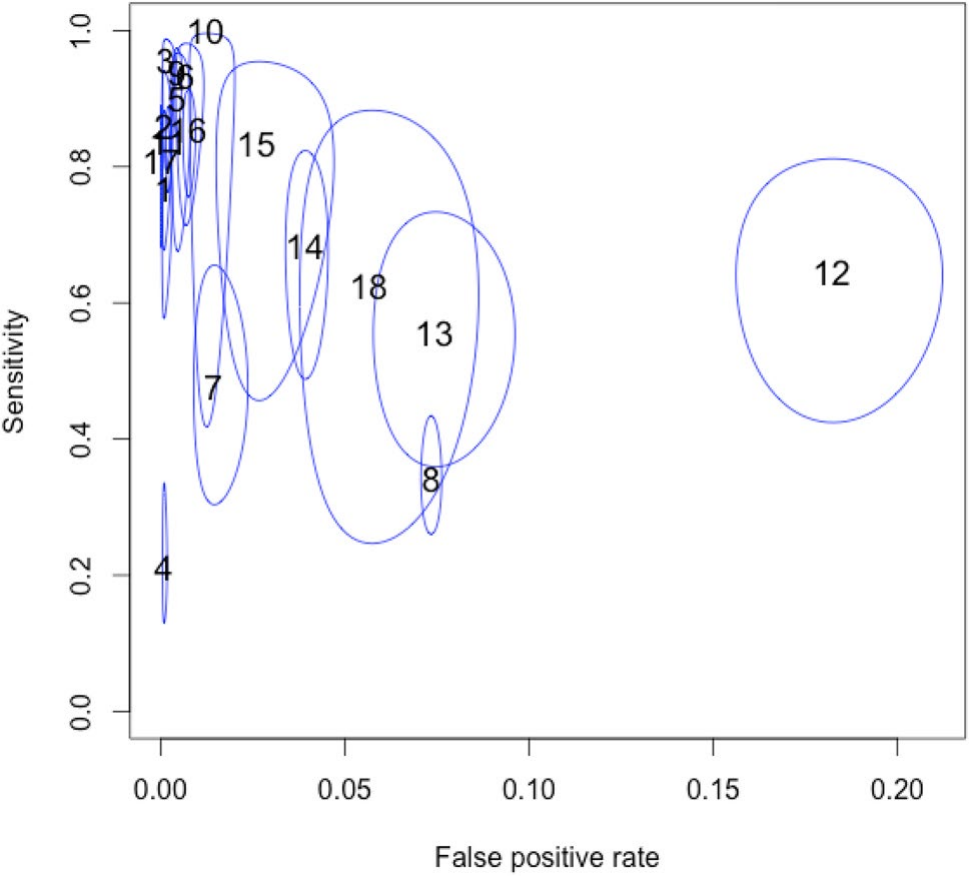
ROC ellipses plot with confidence regions, which describe the uncertainty of the pair of sensitivity and false positive rate. The size of the circles indicates the weight of each study. Studies indicated by study number (see Table 1)

The accuracy of screening tools was evaluated in 14 studies that assessed the test characteristics of various screening tools (18 in all). The pooled *Se* was 0.72 (95% CI 0.61–0.81) and the *Sp* was 0.98 (95% CI 0.97–0.99). The positive likelihood ratio (LR+) was 131.27 (95% CI 50.40–344.48) and the negative likelihood ratio (LR−) was 0.22 (95% CI 0.13–0.45). The diagnostic odds ratio (DOR) was 596.09 (95% CI 174.32–2038.34). The positive predictive value (PPV) was 97.78 (95% CI 97.71–97.84) and the negative predictive value (NPV) was 93.13 (95% CI 93.02–93.24). The above is summarized in Table 3, while the corresponding HSROC plot is presented in Fig. 3. The *Se* of each individual study varied between 0.22 and 0.95 whereas the *Sp* ranged from 0.81 to 0.99 (see Table 4).

**Table 3:** Parameters estimated between studies (point estimate = median) both for the entire meta-analysis and for the sub-analysis of nine studies

Parameters	Meta-analysis with all studies selected (N = 18)					Meta-analysis: subgroup of analysis (N = 9)				
	Estimated	*SD*	MC_error	C.I._lower	C.I._upper	Estimated	*SD*	MC_error	C.I._lower	C.I._upper
HSROC THETA^a^	0.86	0.13	< 0.01	0.12	0.60	0.51	0.16	0.01	0.16	0.17
HSROC LAMBDA^b^	2.89	0.13	< 0.01	2.59	2.99	2.90	0.14	< 0.01	2.56	2.99
HSROC Beta^c^	− 0.09	< 0.01	< 0.01	− 0.09	− 0.09	0.38	0.09	0.01	0.20	0.55
σ_α_^d^	1.09	0.21	< 0.01	0.74	1.57	1.07	0.31	0.01	0.59	1.77
σ_θ_^e^	0.51	0.10	< 0.01	0.35	0.75	0.32	0.13	< 0.01	0.14	0.60
*Se* overall	0.72	0.05	< 0.01	0.61	0.81	0.77	0.03	< 0.01	0.69	0.84
*Sp* overall	0.98	< 0.01	< 0.01	0.97	0.99	0.99	< 0.01	< 0.01	0.97	0.99

MC error of each parameter smaller than 10% of its posterior standard deviation

*Se* sensitivity, *Sp* specificity

^a^THETA = the overall mean cut-off value for defining a positive test

^b^LAMBDA = the overall diagnostic accuracy

^c^Beta = the logarithm of the ratio of the standard deviation of test results among patients with the disease and among patients without the disease

^d^σ_α_ = the between-study standard deviation of the difference in means

^e^σ_θ_ = the between-study standard deviation in the cut-off

**Table 4:** Estimates of diagnostic precision and outcomes in single studies

Study	Screening test	THETA^a^ (95% CI)		ALPHA^b^ (95% CI)		Prevalence^c^ (95% CI)		Sensitivity (*Se*) (95% CI)		Specificity (*Sp*) (95% CI)	
		Estimated	*SD*	Estimated	*SD*	Estimated	*SD*	Estimated	*SD*	Estimated	*SD*
Nygren et al. (2012)	M-CHAT	1.31 (1.06–1.56)	0.12	3.95 (3.45–4.46)	0.24	0.01 (< 0.01–0.01)	< 0.01	0.75 (0.63–0.87)	0.06	0.99 (0.99–1)	< 0.01
Nygren et al. (2012)	JOBS	1.16 (0.89–1.41)	0.13	4.21 (3.72–4.72)	0.25	0.01 (< 0.01–0.01)	< 0.01	0.84 (0.72–0.93)	0.05	0.99 (0.99–1)	< 0.01
Nygren et al. (2012)	M-CHAT + JOBS	0.86 (0.58–1.12)	0.13	4.52 (4.02–5.03)	0.25	0.01 (< 0.01–0.01)	< 0.01	0.92 (0.85–0.98)	0.03	0.99 (0.99–1)	< 0.01
Baird et al. (2000)	CHAT	1.99 (1.84–2.15)	0.07	2.58 (2.27–2.86)	0.15	< 0.01 (< 0.01 to < 0.01)	< 0.01	0.22 (0.15–0.31)	0.04	0.99 (0.99–1)	< 0.01
Wigginset al. (2014)	M-CHAT	0.81 (0.53–1.05)	0.13	3.86 (3.37–4.40)	0.26	< 0.01 (< 0.01–0.01)	< 0.01	0.88 (0.77–0.96)	0.05	0.99 (0.99–1)	< 0.01
Wigginset al. (2014)	PEDS + PATH	0.65 (0.39–0.94)	0.13	3.88 (3.33–4.44)	0.28	0.01 (< 0.01–0.01)	< 0.01	0.91 (0.80–0.97)	0.04	0.99 (0.99–1)	< 0.01
Kamio et al. (2014)	M-CHAT_JV	1.15 (0.98–1.35)	0.09	2.28 (1.89–2.64)	0.19	0.02 (0.01–0.03)	< 0.01	0.49 (0.35–0.62)	0.07	0.98 (0.98–0.99)	< 0.01
Stenberg et al. (2014)	M-CHAT	− 0.05 (− 0.14–0.01)	0.05	3.13 (2.97–3.31)	0.09	< 0.01 (< 0.01 to < 0.01)	< 0.01	0.95 (0.93–0.97)	< 0.01	0.92 (0.92–0.93)	< 0.01
Chlebowski et al. (2013)	M-CHAT /YALE SCREENER and STAT	0.76 (0.59–0.91)	0.08	3.98 (3.68–4.30)	0.15	< 0.01 (< 0.01 to < 0.01)	< 0.01	0.90 (0.84–0.95)	0.02	0.99 (0.99–1)	< 0.01
Canal-Bedia et al. (2011)	M-CHAT	0.54 (− 0.01 to − 1.03)	0.26	3.63 (2.63–4.69)	0.52	< 0.01 (< 0.01 to < 0.01)	< 0.01	0.90 (0.68–0.99)	0.09	0.98 (0.98–0.99)	< 0.01
Barbaro and Dissanayake (2010)	SACS	1.06 (0.96–1.16)	0.05	3.90 (3.70–4.10)	0.10	0.01 (< 0.01–0.01)	< 0.01	0.82 (0.77–0.87)	0.02	0.99 (0.99–1)	< 0.01
Inada et al. (2011)	M-CHAT (short version 9, cutoff:1)	0.23 (< 0.01–0.43)	0.10	1.44 (1.02–1.85)	0.20	0.02 (0.01–0.03)	< 0.01	0.69 (0.54–0.83)	0.07	0.81 (0.79–0.84)	0.01
Inada et al. (2011)	M-CHAT (full version)	0.66 (0. 47–0.84)	0.09	1.71 (1.31–2.07)	0.19	0.03 (0.02–0.04)	< 0.01	0.58 (0.43–0.72)	0.07	0.92 (0.91–0.94)	< 0.01
Dereu et al. (2010)	CESDD	0.68 (0.56–0.83)	0.07	2.32 (2.02–2.59)	0.15	< 0.01 (< 0.01 to <0.01)	< 0.01	0.69 (0.58–0.77)	0.05	0.96 (0.95–0.96)	< 0.01
Miller et al. (2011)	ITC + M-CHAT	0.61 (0.27–0.93)	0.17	2.89 (2.23–3.61)	0.34	0.01 (0.01–0.03)	< 0.01	0.81 (0.62–0.96)	0.08	0.97 (0.96–0.98)	< 0.01
Robins et al. (2014)	M-CHAT-R/F	0.78 (0.67–0.91)	0.06	3.53 (3.27–3.79)	0.13	< 0.01 (< 0.01 to < 0.01)	< 0.01	0.84 (0.78–0.90)	0.03	0.99 (0.99–1)	< 0.01
Honda et al. (2005)	YACHT-18	1.58 (1.41–1.75)	0.08	4.27 (4.00–4.56)	0.14	< 0.01 (< 0.01–<0.01)	< 0.01	0.71 (0.63–0.79)	0.04	0.99 (0.99–1)	< 0.01
Baranek (2015)	M-CHAT	0.68 (0.31–1.33)	0.18	1.99 (1.27–2.71)	0.37	0.01 (< 0.01–0.01)	< 0.01	0.62 (0.35–0.85)	0.13	0.94 (0.92–0.96)	< 0.01

*Se* sensitivity, *Sp* specificity

^a^THETA = the overall mean cut-off value for defining a positive test

^b^ALPHA = the ‘accuracy parameter’ measures the difference between TP and FP within-study parameters

^c^Prevalence within-study parameters

### Exploration of Heterogeneity

A considerable degree of heterogeneity in sensitivities was observed (Q = 337.62, df = 17.00, p < 0.001) and specificities (Q = 30901.50, df = 17.00, p < 0.001). The heterogeneity in test accuracy between studies may be due to differences in cut-offs utilized in different studies, among other factors (Doebler et al. 2012). To delve deeper into the understanding of these results, we evaluated the confidence intervals which describe the relationship between the psychometric properties. The ROC ellipse plots of the confidence intervals in Fig. 3 shows the studies responsible for high levels of heterogeneity, how cut-off values vary, and how they demonstrate moderate negative correlations between sensitivities and False Positive rates (*r*_*s*_ = − 0.355), that is, if *Se* tends to decrease when *FP* rate increases.

According to this analysis, study 18 (Baranek 2015), study 14 (Dereu et al. 2010), studies 12 and 13 (Inada et al. 2011) and study 15 (Miller et al. 2011) show the largest confidence intervals both for *Se* and *FP* rate, and study 4 (Baird et al. 2000), study 10 (Canal-Bedia et al. 2011), study 7 (Kamio et al. 2014) and study 8 (Stenberg et al. 2014) indicate large confidence intervals only in *Se*.

The SROC curve summarizes the relationship between *Se* and (1 − *Sp*) across studies, taking into account the between-study heterogeneity. We constructed a SROC curve using all studies selected; see Fig. 3. It is worth noting that it is a significant graphical tool for understanding how the diagnostic accuracy of the different test depends on the different cut-off (Doebler et al. 2012).

**Fig. 4:**
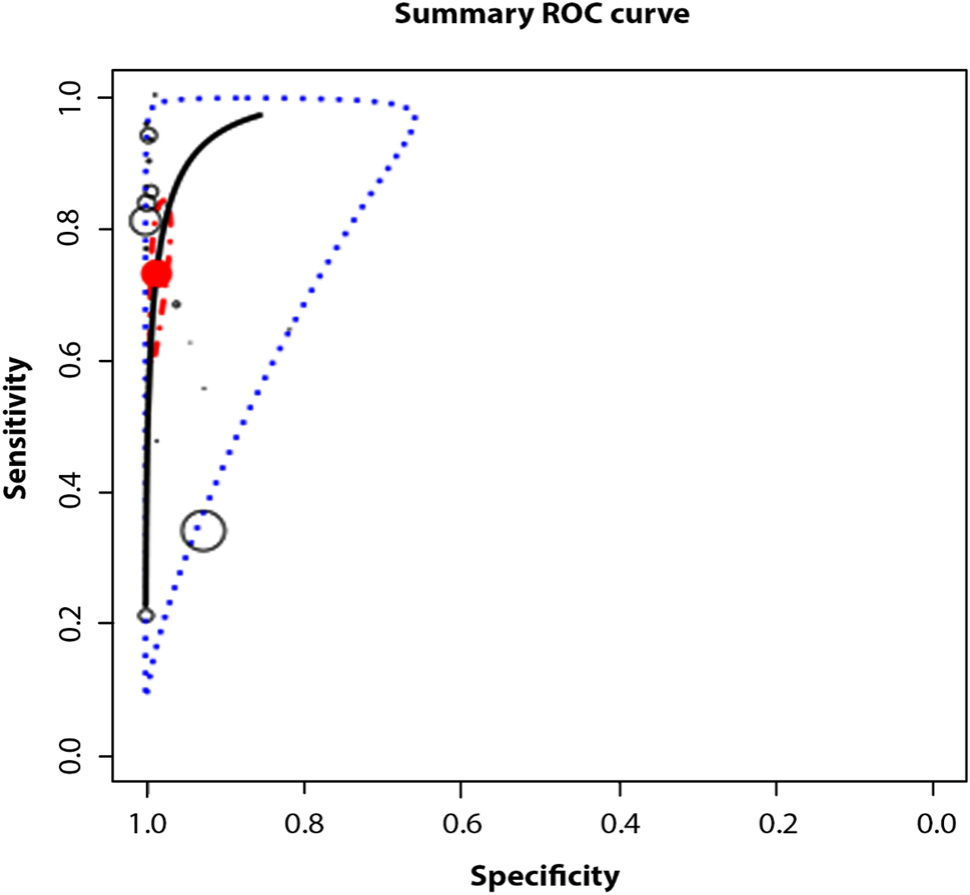
Hierarchical summary receiver operating characteristic curve (HSROC) plot shows test accuracy (using all studies selected). According to Schiller and Dendukuri (2015) individual studies are represented by round circles. The size of the circles is proportional to the number of patients included in the study, the height of ovals indicates the number of affected individuals and the width indicates the number of non-affected individuals. The filled red circle is the pooled sensitivity and specificity across the studies taking into account the between-study heterogeneity. The blue dotted-curve defines the 95% prediction region. The red dot-dashed-curve marks the boundary of the 95% credible region for the pooled estimates

As Fig. 4 shows, the prediction region covers a larger range of *Se* than *Sp*. This may be due to the fact that most of the studies had a considerably larger number of participants with screen negative results compared to screen positive results, leading to greater sampling variability when we estimated *Se* vs. *Sp*. The figure also demonstrates an asymmetry of the test performance measures towards a higher *Sp* with higher variability of *Se*, providing indirect proof of some threshold variability. The figure also shows how when the threshold is increased then *Se* is decreased but *Sp* is increased.

The posterior predictive value of *Se* was 0.71 (95% CI 0.22–1) with a standard error of 0.23 and that of *Sp* was 0.98 (95% CI 0.81–1) with a standard error of 0.07.

### Subgroup of Analysis

A large degree of heterogeneity was observed. Heterogeneity may be due to different factors (Macaskill et al. 2010; Trikalinos et al. 2012). In order to investigate the source of heterogeneity in the current sample, we followed recommendations of these authors and conducted analyses using a subgroup of studies. The new meta-analysis excluded the following studies, based on graphical analysis and the Cochran Q test (p > 0.1): Study 4 (Baird et al. 2000), Study 7 (Kamio et al. 2014), Study 8 (Stenberg et al. 2014), Study 10 (Canal-Bedia et al. 2011), Studies 12 and 13 (Inada et al. 2011), Study 14 (Dereu et al. 2010), Study 15 (Miller et al. 2011), and Study 18 (Baranek 2015).

Regarding the estimations between study parameters, subgroup analysis demonstrated that *Se* was increased because the pooled sensitivity was 0.77 (95% CI 0.69–0.84), and the *Sp* was 0.99 (95% CI 0.97–0.99). The posterior predictive *p*-value of *Se* was 0.81 (95% CI 0.39–1) and *Sp*, 0.97 (95% CI 0.76–1, SD = 0.08).

Parameters estimated between studies by HSROC model are shown in Table 3, which demonstrates how the parameters estimated for the subgroup of analysis are higher results than those obtained for the first meta-analysis. For example, it is of note that standard deviation in the cut-off and standard deviation of the difference in means between studies are decreased.

The estimates for individual studies were grouped by parameters and are shown in Table 5.

**Table 5:** Estimates of diagnostic precision and outcomes in single studies for the sub-analysis of nine studies

Study	Screening test	THETA^a^ (95% CI)		ALPHA^b^ (95% CI)		Prevalence^c^ (95% CI)		*Se* (95% CI)		*Sp* (95% CI)	
		Estimated	*SD*	Estimated	*SD*	Estimated	*SD*	Estimated	*SD*	Estimated	*SD*
Nygren et al. (2012)	M-CHAT	0.82 (0.47–1.14)	0.17	3.56 (3.45–4.46)	0.29	0.01 (< 0.01–0.01)	< 0.01	0.78 (0.65–0.90)	0.06	0.99 (0.99–1)	< 0.01
Nygren et al. (2012)	JOBS	0.65 (0.31–0.98)	0.17	3.93 (3.72–4.72)	0.28	0.01 (< 0.01 -01)	< 0.01	0.86 (0.76–0.94)	0.05	0.99 (0.99–1)	< 0.01
Nygren et al. (2012)	M-CHAT + JOBS	0.34 (-0.03–0.71)	0.19	4.32 (4.02–5.03)	0.33	0.01 (< 0.01–0.01)	< 0.01	0.93 (0.85–0.98)	0.03	0.99 (0.99–1)	< 0.01
Wiggins et al. (2014)	M-CHAT	0.35 (− 0.06 to 0.76)	0.20	3.61 (3.37–4.40)	0.33	< 0.01 (< 0.01–0.01)	< 0.01	0.88 (0.76–0.96)	0.05	0.99 (0.99–1)	< 0.01
Wiggins et al. (2014)	PEDS + PATH	0.24 (− 0.15 to 0.76)	0.20	3.57 (3.33–4.44)	0.36	0.01 (< 0.01–0.01)	< 0.01	0.89 (0.77–0.98)	0.04	0.99 (0.99–1)	< 0.01
Chlebowski et al. (2013)	M-CHAT /YALE SCREENER/STAT	0.24 (0.04–0.42)	0.10	3.87 (3.68–4.30)	0.21	< 0.01 (< 0.01 to < 0.01)	< 0.01	0.91 (0.85–0.95)	0.02	0.99 (0.99–1)	< 0.01
Barbaro and Dissanayake (2010)	SACS	0.60 (0.36–0.81)	0.10	3.56 (3.70–4.10)	0.14	0.01 (< 0.01 to < 0.01)	< 0.01	0.83 (0.78–0.88)	0.02	0.99 (0.99–1)	< 0.01
Robins et al. (2014)	M-CHAT-R/F	0.36 (0.14–0.49)	0.08	3.26 (3.27–3.79)	0.15	< 0.01 (< 0.01 to <0.01)	< 0.01	0.85 (0.80–0.91)	0.03	0.99 (0.99–1)	< 0.01
Honda et al. (2005)	YACHT-18	0.98 (0.66–1.29)	0.16	4.15 (4.00–4.56)	0.20	< 0.01 (< 0.01 to <0.01)	< 0.01	0.81 (0.73–0.89)	0.04	0.99 (0.99–1)	< 0.01

MC error of each parameter smaller than 10% of its posterior standard deviation

*Se* sensitivity, *Sp* specificity

^a^THETA = the overall mean cut-off value for defining a positive test

^b^ALPHA = the ‘accuracy parameter’ measures the difference between TP and FP within-study parameters

^c^Prevalence within-study parameters

**Fig. 5:**
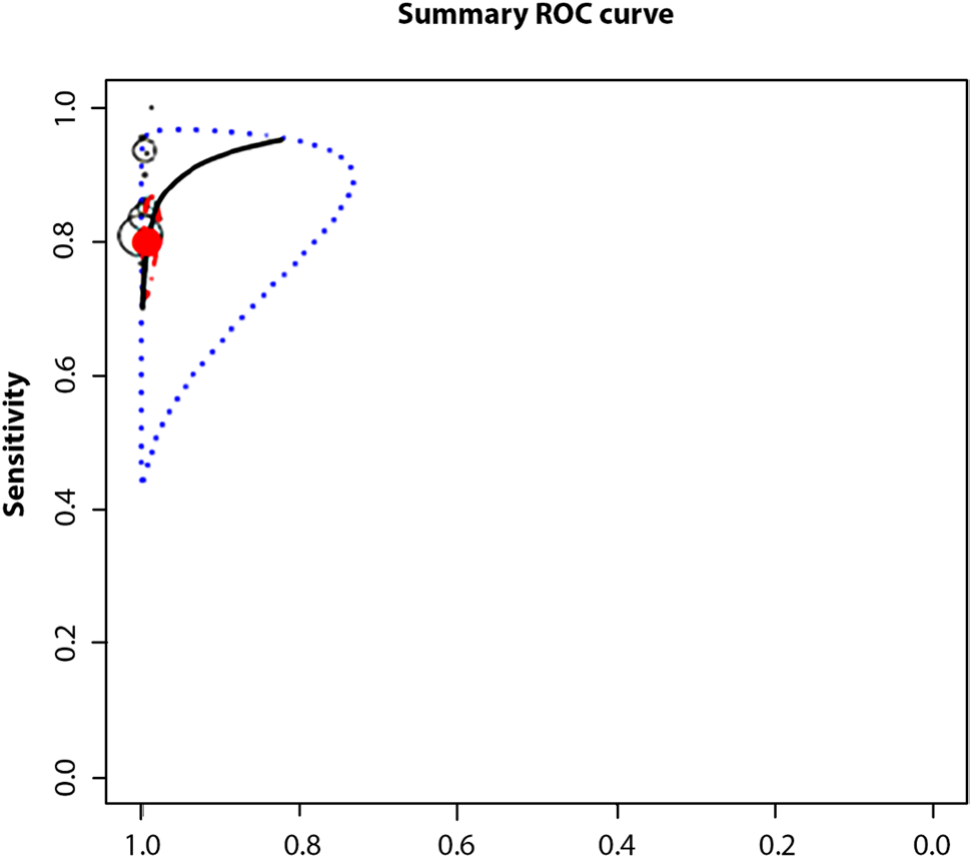
Hierarchical summary receiver operating characteristic curve (HSROC) plot show test accuracy (using subgroup of studies)

Figure 5 shows how the prediction region covers a larger range of *Se* than *Sp* although this is less than in the first meta-analysis. The figure also shows less asymmetry of the test performance and therefore less heterogeneity. This means that the range, which includes the measurements for *Se* and *Sp* is lower than the one shown in Fig. 4.

### Publication Bias

The estimated Egger bias coefficient was 3.21 (95% CI − 0.49 to 6.92) with a standard error of 1.5, giving a *p*-value of 0.08. The test thus suggests evidence that results are not biased by the presence of small-study effects.

## Discussion

Interest in early detection of ASD is increasing, due to the growing evidence that early intervention improves prognosis. Low-risk screening, as part of pediatric primary care, for example, is one of the most widely studied strategies to promote early detection.

Consequently, the information reported from systematic reviews of screening accuracy is valuable, both for research and practice. Different systematic reviews, such as the ones carried out by Daniels et al. (2014) and McPheeters et al. (2016), have represented an important advance with regard to traditional or narrative reviews, which were characterized by a lack of systematization. However, a meta-analysis is a systematic review which also uses statistical methods to analyze the results of the included studies. It is accepted that data from systematic reviews with meta-analyses adds value since the statistical analysis used converts the results of primary studies into a measure of integrated quantitative evidence. This is beneficial both to the scientific community and to the clinicians who use the tools in such meta-analyses.

Meta-analysis of screening studies is a complex but critical approach to examining evidence across measures and scoring thresholds in different populations (Gatsonis and Paliwal 2006). We employed a Bayesian Hierarchical Model (Rutter and Gatsonis 2001), which is robust in adjusting for the imperfect nature of the reference standard of autism tools, in a bivariate meta-analysis of diagnostic test sensitivity and specificity and others psychometric parameters. This kind of meta-analysis statistically compares the accuracy of different diagnostic screening tests and describes how test accuracy varies. Therefore, it is more likely to lead to a ‘gold standard’ than other types of reviews which can be influenced by biases associated with the publication of single studies.

The HSROC model was used to estimate the *screening accuracy* parameters and a summary in each study as functions of an underlying bivariate normal model. This model has been recommended when there is no standard cut-off to define a positive result (Bronsvoort et al. 2010; Dukic and Gatsonis 2003; Macaskill 2004) in order to allow the meta-analytic assessment of heterogeneity between studies while taking into consideration both within- and between-study variability. Furthermore, it is also optimally suited when more information is available, for example, when the studies have reported results from more than one modality (Rutter and Gatsonis 2001) like our case. The advantages of the model have been discussed (Gatsonis and Paliwal 2006; Leeflang et al. 2013; Macaskill 2004; Rutter and Gatsonis 2001) and support its selection in this meta-analysis.

This review included 14 studies that assessed the test characteristics of various screening tools (18 in all) for detecting autism and a subgroup of analysis retaining nine studies that demonstrated lower heterogeneity. Initial findings of the overall meta-analysis show that tools which are used in level 1 ASD screening are accurate at detecting the presence of ASD [pooled sensitivity was 0.72 (95% CI 0.61–0.81)] and highly accurate at detecting a lack of presence of ASD [pooled of specificity was 0.98 (95% CI 0.97–0.99)]. But more importantly, we demonstrate the tools’ performance in identifying autism, DOR 596.09 (95% CI 174.32–2038.34). The clinical utility of the level 1 screening tools reviewed in this study is clear because the pooled positive likelihood ratio (LR+) was 131.27 (95% CI 50.40–344.48) and the negative likelihood ratio (LR−) was 0.22 (95% CI 0.13–0.45). LR+ > 1 indicates the results are associated with the disease. Although those findings are informative to clinicians, it is important to understand the limitations of the last assertion because the accuracy of a *LR* depends upon the quality of the studies that generated the pooled of sensitivity and specificity, therefore data must be interpreted with caution. Finally, the pooled of positive predictive value (PPV) was 97.78 (95% CI 97.71–97.84) and the negative predictive value (NPV) was 93.13 (95% CI 93.02–93.24).

A limitation of this meta-analysis comes from the methodological limitations of the included studies; 55% of the included studies were assessed to have high risk or unclear risk of bias in the quality analysis with QUADAS, particularly in the domains of flow and timing, and in the index test. We recommend that future screening studies include a flowchart with information about the method of recruitment of patients, sample, order of test execution, follow up and other details related to the process to improve replicability and to better inform readers about potential bias.

The second concern is about the heterogeneity of the psychometric data in the included studies. In this respect, according to Doebler et al. (2012), in diagnostic meta-analysis the observed sensitivities and specificities can vary across primary studies and heterogeneity should be assumed in results of this kind of meta-analysis (Macaskill et al. 2010). This assertion has been acknowledged in this work and justifies the choice of the model HSROC, which is a more robust model for addressing heterogeneity compared to some of the other meta-analysis models.

Following the recommendations of Macaskill et al. (2010) and Trikalinos et al. (2012) we conducted a *subgroup of analyses* to assess the pooled *Se* and *Sp* without those studies driving heterogeneity in analyses. The pooled of sensitivity and specificity were improved by the exclusion of these studies. Consequently, the parameters estimated for this set of studies suggested a good performance for ruling out and ruling in ASD since the prior pooled *Se* was 0.77 (95% CI 0.69–0.84, SD = 0.03), *Sp* was 0.99 (95% CI 0.97–0.99; SD ≤ 0.01), the posterior predictive *p-*value of *Se* was 0.81 (95% CI 0.39–1, SD = 0.18), and high specificity was maintained, 0.97 (95% CI 0.76–1, SD = 0.08). The previous data from the posterior predictive *p*-values of *Se* and *Sp* are very important because *the true estimate of Se and Sp* in each study could be found by empirical Bayes estimates (Harbord and Whiting 2009).

One important aspect to bear in mind is that only about 66.6% of all studies showed all the primary outcomes required to populate 2 × 2 contingency tables. Data pertaining to the *Se* were presented in 77.7% of studies, *Sp* in 55.5%, *PPV* in 77.7%, *NPV* in 44.4%, *LR*+ and *LR*− in 22.2% of studies. This leads us to recommend that authors of screening studies include sufficient detail to calculate all psychometric properties to improve the quality of systematic reviews and future meta-analyses. It also would be valuable for authors of future studies to reflect on the question of why there is such a low percentage of primary studies that do provide those data. Some authors use caution in presenting psychometric properties when the negative cases cannot be confirmed to be true negatives. Although this is a notable limitation of cross-sectional screening studies, given that confirmatory evaluations are prohibitive in very large samples, it is likely that the number of truly negative cases greatly outnumbers those cases that will later be identified as false negatives, suggesting that interpreting the TN cell of the 2 × 2 matrix to be “presumed TN” is a reasonable assertion. Looking further at the omission of specific psychometric values, there is a remarkably low percentage of studies that include LR+ and LR−, as well as a number that do not report NPV. LR+ and LR− may not have been commonly included given that they were not emphasized in the American Academy of Pediatrics’ policy statement that highlighted the psychometric properties of Se and Sp. The reduced emphasis on NPV may be due to the fact that predictive value is affected by baserate of the disorder in the sample being studied (such as PPV and NPV may vary dramatically across sampling strategies), whereas Se and Sp are not influenced by base rate. We recommend that future studies report comprehensive psychometrics, in order to promote understanding of the findings. In addition, it is often difficult to ascertain characteristics of the study, study cohort, and technical aspects (Gatsonis and Paliwal 2006). In future studies, a unified approach is necessary in presenting results of screening research to avoid the inconsistency and heterogeneity observed.

The present results suggested improved screening accuracy when meta-analysis was restricted to a subset of studies with reduced heterogeneity (see Table 3 for a comparison of parameters for the complete meta-analysis and the subgroup meta-analysis). The subgroup findings add specific knowledge for clinicians and researchers regarding each tool used for toddler ASD screening.

We have estimated parameters for each study in both meta-analyses (see Tables 4, 5). The results from subgroup analysis suggest that the *Se* of each individual study varied between 0.78 and 0.88. In those tables we also reported other important data, which could be a particular contribution for the clinicians in this field of study, such as the different cut-off points or the ‘accuracy parameter’, which measures the difference between *TP* and *FP* in each study and the prevalence. With respect to prevalence, we can say that it was estimated at or near 1% depending on the studies.

Finally, in the light of the results obtained by computing the summary measures with and without studies (shown as outliers Tables 3, 4, 5) we suggest that the tools used in Level 1 screening are adequate to detect ASD in the 14–36 age range. Thus, we confirm -*in quantitative terms*- the finding of the USPSTF that screening detects ASD.

## Conclusion

A systemic review and meta-analysis of screening tools to detect ASD in toddlers determined that these measures detect ASD with high *Se* and *Sp*. Studies were restricted to low-risk samples in children younger than 3 years old, in order to evaluate the use of these screening tools in primary pediatric care. Given that children who start ASD-specific early intervention before age three have improved outcomes compared to children who go untreated prior to preschool, it is essential to disseminate strategies to improve the identification of the children in need of intervention as young as possible. Consistent with the recommendation of the American Academy of Pediatrics (Johnson et al. 2007) results of the current study show the validity of low-risk screening to identify ASD in children under 3 years old.

### Electronic supplementary material

Below is the link to the electronic supplementary material.

Supplementary material 1 (DOCX 16 KB)

## Appendix 1

### The Search Strategy Described on PubMed was Carried on May 2015

#1 “Autistic Disorder” [Majr] OR “Autistic Disorder” [Title/Abstract] OR “Autistic Disorders” [Title/Abstract] OR “Autism” [Title/Abstract] OR “Child Development Disorders, Pervasive” [Majr] OR “Pervasive Developmental Disorder” [Title/Abstract] OR “Pervasive Developmental Disorders” [Title/Abstract] OR “PDD” [Title/Abstract] OR “Autistic Spectrum Disorder” [Title/Abstract] OR “Autistic Spectrum Disorders” [Title/Abstract] OR “Autism Spectrum Disorder” [Title/Abstract] OR “Autism Spectrum Disorders” [Title/Abstract] OR “ASD” [Title/Abstract]

#2 “Diagnosis” [Mesh:noexp] OR “Diagnosis” [Subheading] OR “Diagnosis” [Title/Abstract] OR “Early Diagnosis” [Mesh:noexp] OR “Early Diagnosis” [Title/Abstract] OR “Detection” [Title/Abstract] OR “Early Detection” [Title/Abstract] OR “Early Identification” [Title/Abstract] OR “Early Intervention” [Title/Abstract] OR “Early Prediction” [Title/Abstract]

#3 “Screening” [Title/Abstract] OR “Early Screening” [Title/Abstract] OR “Mass Screening” [Majr:noexp] OR “Mass Screening/instrumentation” [Majr:noexp] OR “Mass Screening/methods” [Majr:noexp] OR “Mass Screening” [Title/Abstract] OR “Screening Tool” [Title/Abstract] OR “Screening Tools” [Title/Abstract] OR “Screening Test” [Title/Abstract] OR “Screening Instrument” [Title/Abstract] OR “Screening Instruments” [Title/Abstract] OR “Checklist” [MeSH Terms] OR “Checklist” [Title/Abstract] OR “Checklists” [Title/Abstract] OR “Follow-up” [Title/Abstract]

#4 (#2 AND #3)

#5 (#1 AND #4)

#6 “Infant” [MeSH Terms:noexp] OR “Child, Preschool” [MeSH Terms] OR “Infant” [Title/Abstract] OR “Infants” [Title/Abstract] OR “Preschool Child” [Title/Abstract] OR “Preschool Children” [Title/Abstract] OR “Toddler” [Title/Abstract] OR “Toddlers” [Title/Abstract]

#7 (#5 AND #6)

#8 “1992/01/01” [PDAT]: “2015/04/31” [PDAT]

#9 English[Lang]

#10 (#7 AND #8 AND #9)

## Appendix 2

### Definitions for Bio-Statistical Terms that may not be Familiar to Readers

*Cochran Q Statistic for Heterogeneity* is used to determine whether variations between primary studies represent true differences or are due to chance. A *p* value < 0.05 indicates the presence of heterogeneity due to the low statistical strength of Cochran’s Q test.

$$Q=\mathop \sum \nolimits^{} {w_i}{\left( {{T_i} - \bar {T}} \right)^2}$$

*Diagnostic accuracy* relates to the ability of a test to discriminate between the target condition and health. This discriminative ability can be quantified by the measures of diagnostic accuracy: sensitivity and specificity/positive and negative predicative values (PPV, NPV)/likelihood ratio/the area under the ROC curve (AUC)/diagnostic odds ratio (DOR).

*Diagnostic Odds Ratio* measures of the effectiveness of a diagnostic test:

$$DOR=(LR+)/(LR- )=(TP/FN)/(FP/TN).$$

*Egger*’*s test* is a simple linear regression of the magnitude of the effect divided by the standard error over the inverse standard error which verifies whether the Y intercept is statistically significant with p < 0.1.

*Graphical analysis* the starting point for investigation of heterogeneity in diagnostic or screening accuracy reviews often is through visual assessment of study results in forest plots and in ROC space.

*Grey literature* is generally understood to mean literature that is not formally published in accessible sources. It can be another source of bias in meta-analytical studies.

*I*^2^*Measure for Heterogeneity* indicates the percentage of variance in a meta-analysis that is attributable to studies heterogeneity. *I*^2^ values range from 0 to 100%. I^2^ values of 25%, 50%, and 75% are interpreted as low, moderate, and high estimates, respectively:

$${I^2}=\left\{ {\begin{array}{*{20}{c}} {\frac{{Q - \left( {k - 1} \right)}}{1} \times \,100\% }&{to\;Q>k - 1} \\ 0&{to\;Q \leqslant k - 1} \end{array}} \right.$$

*Negative Likelihood Ratio* (*LR*−) shows how much the odd of the target condition is decreased when the test index is negative.

$$LR- =(1 - Se)/Sp$$

*Negative Predictive Value (NPV)* probability of no target condition among patients with a negative index test result.

$$NPV=(TN)/(TN+FN)$$

*Positive Predictive Value (PPV)* probability of target condition among patients who actually have the disease.

$$PPV=TP/(TP+FP)$$

*Positive Likelihood Ratio* (*LR*+) shows how much the odds of the target condition are increased when the test index is positive.

$$LR+=Se/(1 - Sp)$$

Publication bias is the term for what occurs whenever the research that appears in the published literature is systematically unrepresentative of the population of completed studies.

The posterior predictive p-value is a Bayesian alternative to the classical p-value. It is used to calculate the tail-area probability corresponding to the observed value of the statistic.

*p-value* The probability under the assumption of null hypothesis, of obtaining a result equal to or more extreme than what was observed. It shows whether a difference found between groups that are being compared is due to chance.

*Sensitivity (Se)* proportion of positives patients with the target condition who are identified as having the condition.

$$Se=(TP)/(TP+FN)$$

*Specificity (Sp)* proportion of negatives patients without the target condition who are identified as not having the condition.

$$Sp=(TN)/(TN+FP)$$
