# Supplementary material for: Toddler Screening for Autism Spectrum Disorder: A Meta-Analysis of Diagnostic Accuracy
Source: J Autism Dev Disord. 2019 Jan 8;49(5):1837–52. doi: 10.1007/s10803-018-03865-2 (PMC6483963; doi:10.1007/s10803-018-03865-2)
Supplement: Supplementary file 1 — Supplementary material 1 (DOCX 16 KB) [file 10803_2018_3865_MOESM1_ESM.docx]

| Table 1  *Papers excluded*  *methodological quality assessment and data extraction* | |  |
| --- | --- | --- |
| Bibliographic reference | Cause of exclusion |  |
| Dietz, Swinkels, van Daalen, van Engeland, & Buitelaar, 2006 | Excluded from quantitative analysis because its outcomes did not allow us to estimate the necessary data for the analysis model |  |
| Honda et al., 2009 | Quality exclusion criteria: The sample is duplicated. This is more fully explain in Honda (2005) |  |
| Kleinman et al., 2008 | Excluded from quantitative analysis because its outcomes did not allow us to estimate the necessary data for the analysis model |  |
| Pierce et al., 2011 | Quality exclusion criteria: the average age = 12, 54 and there is a paragraph of the paper where the authors argue that the sensitivity and specificity values estimated are not reliable. |  |
| Robins, 2008 | Quality exclusion criteria: The sample is overlapped with Chlebowski (2013) |  |
| VanDenHeuvel, Fitzgerald, Greiner, & Perry, 2007 | Quality exclusion criteria: it does not explain diagnostic measures. |  |
| Wetherby, Brosnan-Maddox, Peace, & Newton, 2008 | Quality exclusion criteria: Age ranges are interrelated in the sample of patients with diagnoses, so we cannot know who the specific patients screened at a certain age are. It is not a population design |  |
|  | | |
